# Conditioned Medium of Bone Marrow-Derived Mesenchymal Stromal Cells as a Therapeutic Approach to Neuropathic Pain: A Preclinical Evaluation

**DOI:** 10.1155/2018/8179013

**Published:** 2018-01-30

**Authors:** Kelly Barbosa Gama, Dourivaldo Silva Santos, Afrânio Ferreira Evangelista, Daniela Nascimento Silva, Adriano Costa de Alcântara, Ricardo Ribeiro dos Santos, Milena Botelho Pereira Soares, Cristiane Flora Villarreal

**Affiliations:** ^1^Instituto Gonçalo Moniz, FIOCRUZ, Rua Waldemar Falcão 121, 40296-710 Salvador, BA, Brazil; ^2^Faculdade de Farmácia, Universidade Federal da Bahia, Barão de Geremoabo s/n, 40170-290 Salvador, BA, Brazil; ^3^Centro de Biotecnologia e Terapia Celular, Hospital São Rafael, Avenida São Rafael 2152, 41253-190 Salvador, BA, Brazil; ^4^National Institute of Science and Technology for Regenerative Medicine, Rio de Janeiro, RJ, Brazil

## Abstract

Neuropathic pain is a type of chronic pain caused by injury or dysfunction of the nervous system, without effective therapeutic approaches. Mesenchymal stromal cells (MSCs), through their paracrine action, have great potential in the treatment of this syndrome. In the present study, the therapeutic potential of MSC-derived conditioned medium (CM) was investigated in a mouse model of neuropathic pain induced by partial sciatic nerve ligation (PSL). PSL mice were treated by endovenous route with bone marrow-derived MSCs (1 × 10^6^), CM, or vehicle. Gabapentin was the reference drug. Twelve hours after administration, neuropathic mice treated with CM exhibited an antinociceptive effect that was maintained throughout the evaluation period. MSCs also induced nonreversed antinociception, while gabapentin induced short-lasting antinociception. The levels of IL-1*β*, TNF-*α*, and IL-6 were reduced, while IL-10 was enhanced on sciatic nerve and spinal cord by treatment with CM and MSCs. Preliminary analysis of the CM secretome revealed the presence of growth factors and cytokines likely involved in the antinociception. In conclusion, the CM, similar to injection of live cells, produces a powerful and long-lasting antinociceptive effect on neuropathic pain, which is related with modulatory properties on peripheral and central levels of cytokines involved with the maintenance of this syndrome.

## 1. Introduction

Neuropathic pain is a progressive nervous system disease initiated by a primary lesion or dysfunction in the nervous system, commonly caused by a trauma, infection, or ischemia. This chronic syndrome is characterized by abnormal sensory symptoms, such as spontaneous pain and an increased response to painful (hyperalgesia) and innocuous (allodynia) stimuli [1–3]. Despite the great morbidity, social cost, and negative impact on quality of life, the neuropathic pain has limited therapeutic options, because the available analgesic drugs appear to be relatively ineffective in controlling this pain state [4]. Up to now, no drug is available to restore nerve function and the usual therapeutic strategies for neuropathic pain aim only palliative analgesic effects [5].

Thus, the successful control of neuropathic pain is linked to the establishment of new disease-modifying therapeutic approaches. In this context, cell-based therapies represent a promising alternative. Recently, cellular therapies have been considered as a potential successful approach in treating diseases and injuries of the nervous system, for which currently no effective treatment strategies are available [6–8]. Stem cells have been shown to exert neuroprotective and regenerative potential in the damaged nervous system [8–10]. Recent studies have indicated mesenchymal stromal cells (MSCs) as a promising category of stem cells having neuroregenerative properties. The effects of mesenchymal cells are mainly related to their ability to produce neurotrophic factors that may support neuronal survival and induce the proliferation and mobilization of endogenous neural stem cell in the sites of nervous system damage [8, 11, 12]. In fact, contrary to the early paradigm of cell replacement and differentiation as a therapeutic mechanism of action, evidence is growing that the disease-modulating activity of MSCs is due to their ability to secrete factors that exert beneficial impact on the damaged tissue [13]. These secretions include a broad spectrum of factors, collectively referred as secretome and extracellular vesicles containing proteins and functional RNAs, which can be found in the medium where the stem cells are cultured. Recent studies have showed that this medium obtained from MSC cultures, named conditioned medium, may cause tissue repair and therapeutic effects in diseases and injuries of the nervous system [14–17]. This approach offers numerous possibilities for therapeutic development based on products secreted by MSCs, overcoming the limitations and risks associated with the cell-based therapeutics. Conditioned medium from MSCs can be manufactured, freeze-dried, packaged, and transported, which are fundamental characteristics to enable its future production as pharmaceuticals for regenerative medicine [18].

Similar to other diseases of the nervous system, neuropathic pain appears to be amenable to stem cell therapy [19–21]. The strategy of cell transplantation for the treatment of pain is focused on cell-based analgesia and neural repair, so that stem cells can represent not only a pain treatment but also a way for repairing the damaged nervous system. In fact, cell therapy for pain neurorestorative treatment is a new concept for pain control along with neural repair [22]. On the other hand, some researchers have proposed that not the regenerative effects of stem cells but their interaction with damaged microenvironment resident cells is determining to the beneficial effects of these cells in neuropathic conditions [21]. Regardless of the action mechanism, a key therapeutic effect of neuroprotective, neurotrophic, neuroregenerative, and immunomodulatory substances secreted by stem cells has undoubtedly been demonstrated in neuropathic pain [22–24]. Considering this scenario, in the present study, the effects of systemic administration of conditioned medium from bone marrow-derived MSCs on pain-like behavior and neuromodulators pathways underlying the maintenance of neuropathic pain were evaluated, and compared to the MSC-induced effects, in a mouse model of neuropathy.

## 2. Materials and Methods

### 2.1. Bone Marrow-Derived Mesenchymal Cell (MSC) Culture and Conditioned Medium Preparation

Mesenchymal stem cells were obtained from the bone marrow of femurs and tibias of mice. Bone marrow collected samples were diluted in Dulbecco's modified Eagle's medium (DMEM; Gibco, Carlsbad, CA, USA), and the fraction with mononuclear cells was obtained by Ficoll-Hypaque gradient (Sigma, St. Louis, MO, USA), after centrifugation at 400 ×g for 30 minutes at 20°C. The interface containing mononuclear cells was collected in individualized tubes and washed twice in incomplete DMEM. Mononuclear cells were resuspended in DMEM medium supplemented with 2 mM L-glutamine, 1 mM sodium pyruvate, 50 *μ*g/ml gentamycin, and 10% fetal bovine serum (all reagents were purchased from Sigma) and cultured at a density of 10^5^ cells/cm^2^ in polystyrene plates. Cell cultures were maintained at 37°C with 5% CO_2_. The cells were expanded during approximately 5 passages, and when 90% confluence was reached, cells were detached using 0.25% trypsin (Invitrogen/Molecular Probes, Eugene, OR, USA) and expanded in new culture bottles (9 × 10^3^ cells/cm^2^). The identity of MSCs was confirmed on the basis of morphological criteria, plastic adherence, and specific surface antigen expression: CD90(+), CD44(+), Sca-1(+), CD45(−), CD34(−), and CD11b(−). Differentiation ability of MSCs was also evaluated after induction using specific media, as previously described [25]. Oil red, alizarin red, and Alcian blue stainings (Sigma-Aldrich) were used to assess adipogenic, osteogenic, and chondrogenic differentiation.

Conditioned medium (CM) was obtained from MSC cultures (5 passage), as previously described [16]. MSCs (7 × 10^6^) were washed 3 times with phosphate-buffered saline (PBS) and transferred to a serum-free DMEM culture medium during 24 h. Then, CM were concentrated 15 times by centrifugation at 4000*g* for 15 min at 13°C, using ultrafiltration units (Amicon Ultra-PL 10, Millipore, Bedford, MA, USA). Filter units were used only one time to avoid membrane saturation. Concentrated CM were then sterilized on 0.22 *μ*m filters (Millipore) and stored at −80°C until use. CM was divided into aliquots of 700 *μ*l before freezing to avoid repeated freeze/thaw cycles. The mean protein concentration of CM was of 1.5–1.8 mg/ml, and there was no difference between fresh and frozen CM. Serum-free DMEM, centrifuged and filtered, was used as control medium (vehicle group).

### 2.2. CM Characterization by Protein Array

Aiming to obtain a broad view of molecules present in CM, it was analyzed for a panel of specified proteins using an antibody array. The relative expression levels of 111 soluble mouse proteins was determined in CM using the Proteome Profiler Mouse XL Cytokine Array (R&D Systems, Minneapolis, MN, USA), according to the manufacturer's instructions. Spot pixel densities on developed X-ray film was collected and analyzed using a transmission-mode scanner and image analysis software. Data are represented as the mean spot pixel density subtracted from the averaged background signal. Antibody arrays were performed on 3 distinct CM samples.

### 2.3. Animals

Experiments were performed on male C57Bl/6 mice (20–25 g) obtained from the Animal Facilities of Instituto Gonçalo Moniz/FIOCRUZ (Brazil). Animals were housed in temperature-controlled rooms (22–25°C), under a 12 : 12 h light-dark cycle, with access to water and food ad libitum. All behavioral tests were performed between 8:00 a.m. and 5:00 p.m., and animals were only tested once. Animal care and handling procedures were in accordance with the National Institutes of Health guide for the care and use of Laboratory animals (NIH, 8023) and the Institutional Animal Care and Use Committee FIOCRUZ (CPqGM 025/2011). Every effort was made to minimize the number of animals used and to avoid any discomfort [26].

### 2.4. Neuropathic Pain Model: Partial Sciatic Nerve Ligation (PSL)

Under deep anesthesia with 2,2,2-tribromoethanol (Sigma Chemical Company, St. Louis, MO, USA) and aseptic conditions, the left sciatic nerve was exposed at high-thigh level and partially ligated as previously described [27]. Briefly, the nerve was carefully freed from surrounding connective tissues and was fixed in its place by pinching the epineurium on its dorsal aspect. An 8-0 silk suture was inserted into the nerve and tightly ligated so that the dorsal 1/3–1/2 of the nerve thickness was trapped in the ligature. The sciatic nerve in the sham group was exposed but left intact. The wound was closed in layers and then the animals were then placed back to their individual cages after recovering from anesthesia in a warm incubator [28].

### 2.5. Assessment of PSL-Induced Pain-Like Behaviors

Pain-like behaviors were assessed for examination of the neuropathic pain state in mice, before (baseline) and daily after PSL surgery. Behavioral tests were done without knowing to which experimental group each mouse belonged.

Withdrawal threshold to mechanical stimulation was measured with von Frey filaments (Stoelting; Chicago, IL, USA). In a quiet room, mice were placed in acrylic cages (12 × 10 × 17 cm) with wire grid floor which allowed full access to the ventral aspect of the hindpaws, 40 min before the beginning of the test. A logarithmic series of 9 filaments were applied to the plantar surface of the ipsilateral hindpaw to determine the threshold stiffness required for 50% paw withdrawal according to the nonparametric method of Dixon, as described by Chaplan and collaborators [29, 30]. A positive response was characterized by the removal of the paw followed by clear flinching movements. The development of sensorial neuropathy was characterized by mechanical allodynia, indicated by the reduction of the paw withdrawal threshold (in grams).

Withdrawal threshold to heat stimulation was determined using the Plantar Test (Hargreaves Apparatus, Ugo Basile Biological Instruments, Gemonio, Italy) as previously described [31]. Similar to the von Frey test, mice underwent an acclimatization period before the beginning of the test. An infrared light source was located under the glass floor and positioned at the center of the hindpaw of mice. On paw withdrawal, a photo-cell automatically shut off the heat source and recorded the time to withdrawal. To avoid thermal injury, there was an upper cutoff limit of 20 s after which the heating was automatically terminated. The stimulation was applied three times with an interval of at least 5 min. The averaged threshold from these three trials was recorded as the thermal nociception threshold [28]. Thermal hyperalgesia was indicated by the reduction of the paw withdrawal threshold (in seconds).

### 2.6. Motor Function Assay

To evaluate the motor performance, mice were submitted to the rotarod test, as previously described [32]. The rotarod apparatus (Insight, Ribeirão Preto, Brazil) consisted of a bar with a diameter of 3 cm, subdivided into five compartments. The bar rotated at a constant speed of 8 revolutions per min. The animals were selected 24 h previously by eliminating those mice that did not remain on the bar for two consecutive periods of 120 s [26]. At the test day, mice from different experimental groups were placed on a rotating rod and the resistance to falling was measured up to 120 s. Mice treated with diazepam (10 mg/kg; Cristália, Itapira, Brazil), the reference drug of the test, were placed on a rotating rod 1 h after treatment. The results are expressed as the average time (s) the animals remained on the rotarod in each group.

### 2.7. Experimental Design

Mice were divided into the following groups (*n* = 6): naïve, sham neuropathic pain (sham), neuropathic pain plus control medium treatment (vehicle), neuropathic pain plus MSC treatment (MSCs), neuropathic pain plus CM treatment (CM), and neuropathic pain plus gabapentin treatment (gabapentin).

Nociceptive tests (von Frey and plantar test) were performed at baseline and daily after the PSL surgical procedure. Seven days after PSL, and after the establishment of behavioral neuropathic pain as assessed by nociceptive tests, the animals received the treatments. Mice from MSCs group were transplanted by tail vein injection with 1 × 10^6^ cells/mouse in a final volume of 100 *μ*l. Mice from CM group received, via the tail vein, 100 *μ*l of conditioned medium from MSCs (1 × 10^6^). The vehicle group received an endovenous injection (100 *μ*l) of serum-free DMEM, centrifuged and filtered (control medium). Seven days after PSL the animals underwent a twice-daily gabapentin (70 mg/kg; Pfizer, São Paulo, Brazil) oral treatment for six consecutive days. Gabapentin dose and frequency of administration was chosen based on previously published data [33].

Twenty-one days after PSL surgery, 6 mice from sham, vehicle, MSCs, and CM groups were sacrificed for biological sampling. Naïve group was included for biological sampling aiming to show the cytokine profile of mice without any manipulation. Motor performance and body weight was recorded once weekly for general toxicity assessment, starting from the baseline behavioral tests through the end of the experimental period.

### 2.8. Cytokine Measurement by ELISA

For the measurement of cytokine levels, the spinal cord, and sciatic nerve were collected at day 21 after PSL, in mice terminally anesthetized with halothane from each experimental group. The L4-L5 spinal segments and 1 cm sciatic nerve sample containing the lesion site (or comparable region of sham-operated mouse) were removed and rapidly frozen and stored at −80°C. Frozen tissues were later homogenized in ice cold phosphate-buffered saline (PBS; 100 mg tissue/ml) to which 0.4 M NaCl, 0.05% Tween 20, and protease inhibitors (0.1 mM PMSF, 0.1 mM benzethonium chloride, 10 mM EDTA, and 20 KI aprotinin A/100 ml) were added (Sigma). The samples were centrifuged for 10 minutes at 3000*g*, and a supernatant aliquot was frozen at −80°C for later quantification. Tumor necrosis factor-*α* (TNF-*α*), interleukin-1*β* (IL-1*β*), interleukin-6 (IL-6), and interleukin-10 (IL-10) levels were estimated using commercially available immunoassay ELISA kits for mice (R&D System, Minneapolis, MN, USA), according to the manufacturer's instructions. The results are expressed as picograms of cytokine per milligram of protein [26].

### 2.9. Data Analysis

All data are presented as means ± standard error of the mean (SEM) of measurements made on six animals in each group. Behavioral data were analyzed using two-way ANOVA (group and time) followed by Bonferroni's multiple comparisons. Remaining data were analyzed using one-way ANOVA followed by Tukey's posttest. All data were analyzed using the Prism 5 computer software (GraphPad, San Diego, CA, USA). Statistical differences were considered to be significant at *p* < 0.05.

## 3. Results

### 3.1. Effects of CM on Pain-Like Behaviors of Neuropathic Mice

**Figure 1:**
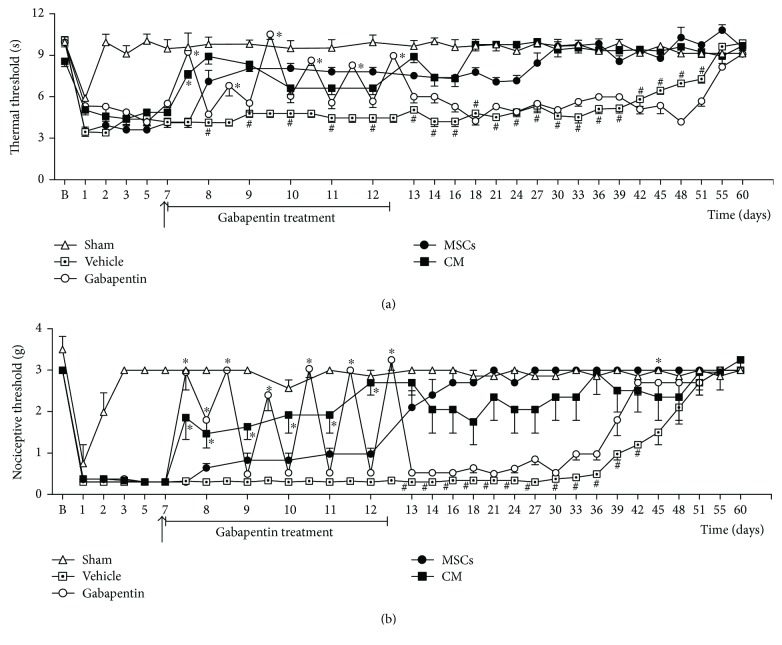
Effect of the conditioned medium from MSCs on PSL-induced neuropathic pain-like behaviors. The nociceptive thresholds were assessed in the ipsilateral paw of each mouse before (b) and after the PSL surgery, performed at time zero. (a) Thermal nociceptive threshold: the axis of ordinates represents the time (seconds) the animal takes to withdraw its paw. (b) Mechanical nociceptive thresholds: ordinates represent the filament weight (g) in which the animal responds in 50% of presentations. Sham group represents mice without neuropathy, in which the sciatic nerve was exposed but left intact. Seven days after PSL mice were treated (arrow) by endovenous route with bone marrow-derived mesenchymal cells (MSCs; 1 × 10^6^/100 *μ*l), conditioned medium from MSCs (CM; 100 *μ*l) or vehicle (control medium; 100 *μ*l). Gabapentin (70 mg/kg), the reference drug, was administered twice daily by oral route for six consecutive days (7 to 12 day). For gabapentin group, nociceptive threshold evaluations were made one hour before and one hour after the first treatment day. Data are expressed as means ± SEM; *n* = 6 mice per group. ^∗^Significantly different from the vehicle-treated group (*p* < 0.05); ^#^significantly different from the MSC and CM groups (*p* < 0.05). Two-way ANOVA followed by the Bonferroni's test.

**Figure 2:**
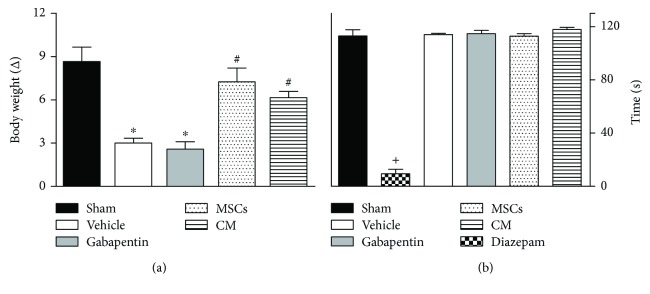
Effects of the conditioned medium from MSCs on motor function and body weight of neuropathic mice. Bar graphs representing (a) the body weight variation of mice from different experimental groups at the end of the experimental period (60 days) and (b) the run time on the rotarod 24 h after treatments. Sham group represents mice without neuropathy, in which the sciatic nerve was exposed but left intact. Seven days after PSL mice were treated by endovenous route with bone marrow-derived mesenchymal cells (MSCs; 1 × 10^6^/100 *μ*l), conditioned medium from MSCs (CM; 100 *μ*l) or vehicle (control medium; 100 *μ*l). Gabapentin (70 mg/kg), the reference drug, was administered twice daily by oral route for six consecutive days. Mice treated with diazepam (10 mg/kg), the reference drug of the rotarod test, were tested 1 h after treatment. Data are reported as means ± SEM; *n* = 6 mice per group. ^∗^Significantly different from the sham group (*p* < 0.01); ^#^significantly different from the vehicle-treated group (*p* < 0.05); ^+^significantly different from the remaining groups (*p* < 0.001). ANOVA followed by Tukey's multiple comparison test.

The therapeutic potential of the CM was evaluated in an established PSL-induced painful neuropathy model. Behavioral testing was performed at baseline and daily after the PSL surgical procedure, and the antinociceptive activity was expressed as reduction of pain-like behaviors. Gabapentin was used as the gold standard drug. PSL surgery induced sensorial neuropathy associated with thermal hyperalgesia and mechanical allodynia in mice without causing motor impairment (Figures 1 and 2(b)). Behavioral signs of sensorial neuropathy were evident 1 day after surgery. Thermal hyperalgesia persisted 51 days (*p* < 0.05), while mechanical allodynia persisted 45 days (*p* < 0.05) after PSL surgery. To determine whether CM induces therapeutic effects in neuropathic states, neuropathic mice were treated with CM, MSCs, or vehicle seven days after PSL surgery, when the sensorial neuropathy was fully stablished. Twelve hours after administration, neuropathic mice treated with CM exhibited antinociceptive effect to thermal and mechanical stimuli (Figure 1; *p* < 0.01). The CM-induced antinociceptive effect was progressive, peaking 11 days after treatment, when a complete reversion of the thermal hyperalgesia was achieved (*p* < 0.001) and maintained throughout the evaluation period (Figure 1(a)). The CM treatment also induced a long-lasting reduction of the mechanical allodynia, from 12 hours until 35 days after administration (Figure 1(b)). Twenty-four hours after MSCs transplantation, neuropathic mice exhibited antinociceptive effect against thermal stimuli, peaking 20 days after treatment (Figure 1(a); *p* < 0.01). The MSC treatment reverted the mechanical allodynia of neuropathic mice from 7 days after administration until the end of the evaluation period (Figure 1(b); *p* < 0.001). The antinociceptive effects of CM was next compared to that of gabapentin, the gold standard to the clinical control of neuropathic pain. Gabapentin (70 mg/kg) was orally administered to mice, twice a day, for six consecutive days starting at day 7. Gabapentin decreased the thermal hyperalgesia and mechanical allodynia in neuropathic mice, but this effect was completely reverted 12 hours after administration (Figure 1; *p* < 0.001). Twelve hours after the last oral administration, gabapentin-treated neuropathic mice exhibited nociceptive thresholds similar to that of vehicle-treated neuropathic mice.

### 3.2. Effects of CM on Motor Performance and General Toxicity in Mice

In order to monitor well-being, mice were daily observed and once weekly weighed throughout the experiment. All mice survived until the end of study. The assessment of body weight changes showed that vehicle-treated mice submitted to PSL exhibited less body weight gain through the experimental period relative to sham mice (Figure 2(a); *p* < 0.001). PSL mice treated with gabapentin also exhibited less body weight gain. By contrast, in CM or MSC-treated mice after PSL the weight gain was similar to that of sham mice. Motor deficit and neurological dysfunctions were discarded, since endovenous administration of CM or MSCs did not affect the motor performance of the mice in the rotarod test (Figure 2(b)). As expected, the central nervous system depressant diazepam (10 mg/kg, intraperitoneal route) reduced the time of mice on the rotarod, after 1 h of treatment with this standard drug (*p* < 0.001).

### 3.3. Effects of CM on Cytokine Levels in Sciatic Nerve and Spinal Cord of Neuropathic Mice

**Figure 3:**
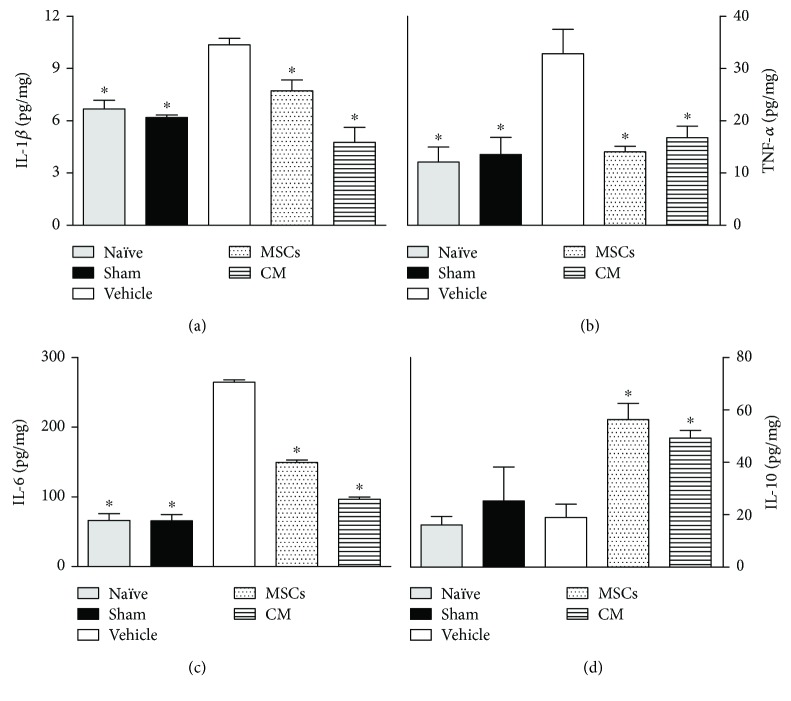
Modulatory effects of the conditioned medium from MSCs on cytokines sciatic nerve levels of neuropathic mice. The naïve group consists of mice that did not receive any experimental manipulation, while the sham group represents mice in which the sciatic nerve was exposed but left intact. Seven days after PSL, mice were treated by endovenous route with bone marrow-derived mesenchymal cells (MSCs; 1 × 10^6^/100 *μ*l), conditioned medium from MSCs (CM; 100 *μ*l), or vehicle (control medium; 100 *μ*l). Panels shows the sciatic nerve levels of (a) interleukin-1*β* (IL-1*β*), (b) tumor necrosis factor-*α* (TNF-*α*), (c) interleukin-6 (IL-6), and (d) interleukin-10 (IL-10), determined in ipsilateral sciatic nerve samples by ELISA at day 21 after the PSL surgery. The results are expressed as picograms of cytokine per milligram of protein. Data are expressed as means ± SEM; *n* = 6 mice per group. ^∗^Significantly different from the vehicle group (*p* < 0.05). ANOVA followed by Tukey's multiple comparison test.

**Figure 4:**
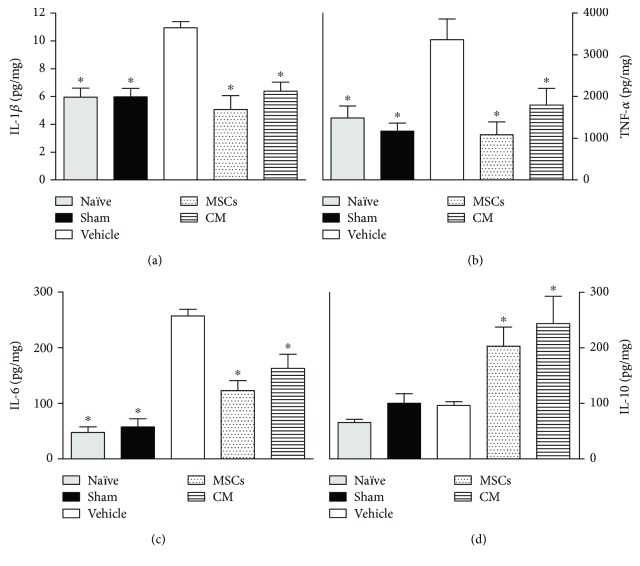
Modulatory effects of the conditioned medium from MSCs on cytokines spinal cord levels of neuropathic mice. The naïve group consists of mice that did not receive any experimental manipulation, while the sham group represents mice in which the sciatic nerve was exposed but left intact. Seven days after PSL mice were treated by endovenous route with bone marrow-derived mesenchymal cells (MSCs; 1 × 10^6^/100 *μ*l), conditioned medium from MSCs (CM; 100 *μ*l), or vehicle (control medium; 100 *μ*l). Panels shows the spinal cord levels of (a) interleukin-1*β* (IL-1*β*), (b) tumor necrosis factor-*α* (TNF-*α*), (c) interleukin-6 (IL-6), and (d) interleukin-10 (IL-10), determined in L4-L5 spinal segments by ELISA at day 21 after the PSL surgery. The results are expressed as picograms of cytokine per milligram of protein. Data are expressed as means ± SEM; *n* = 6 mice per group. ^∗^Significantly different from the vehicle group (*p* < 0.05). ANOVA followed by Tukey's multiple comparison test.

The dysregulation of cytokines at sites of both the central and peripheral nervous systems is a key event in the development and maintenance of neuropathic pain. Considering that a single CM administration induced complete reversion of pain-like behaviors in neuropathic mice, a possible modulatory action of CM on cytokine production during neuropathy was next evaluated. The levels of cytokines were evaluated 21 days after sciatic nerve surgery, that is, 14 days after treatments. This time point was chosen based on behavioral results, indicating a slight maximum antinociceptive effect of CM at this time. Data obtained by ELISA analysis shows that vehicle-treated PSL mice exhibited upregulation of IL-1*β* (Figures 3(a) and 4(a)), TNF-*α* (Figures 3(b) and 4(b)), and IL-6 (Figures 3(c) and 4(c)) in both the spinal cord and sciatic nerve, relative to naïve and sham mice (*p* < 0.05). The levels of IL-1*β*, TNF-*α*, and IL-6 were reduced, while IL-10 was enhanced in the sciatic nerve of neuropathic mice treated with CM or MSCs (Figure 3; *p* < 0.05). Likewise, CM or MSC treatments reduced the IL-1*β*, TNF-*α*, and IL-6 levels in the spinal cord of PSL mice (Figure 4; *p* < 0.05). The spinal levels of IL-10 in neuropathic mice were enhanced by both the CM and MSCs treatments.

### 3.4. Analysis of the CM Secretome by Protein Array

**Figure 5:**
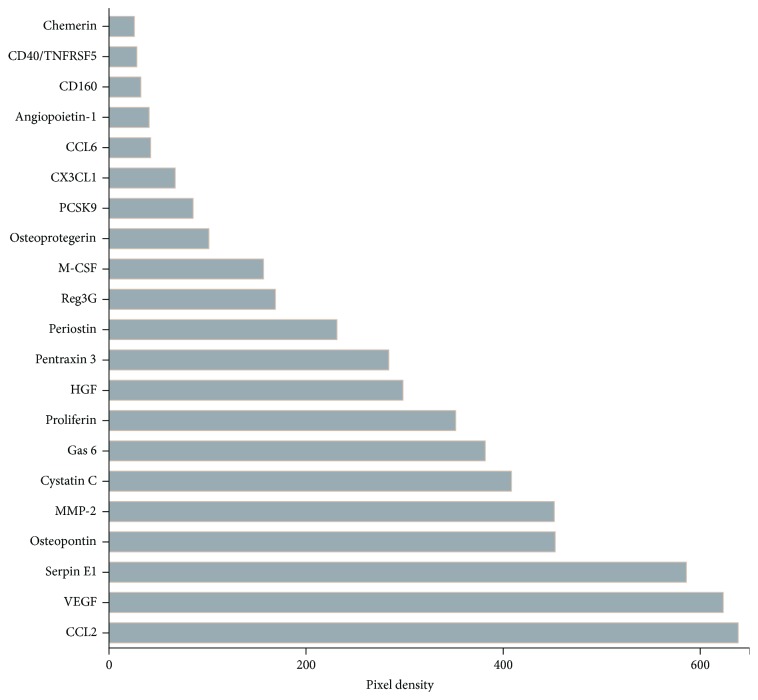
Factors detected in conditioned medium from MSCs. Antibody arrays against 111 specified proteins were performed on 3 different samples of CM and revealed the presence of chemokines, cytokines, binding proteins, enzymes, and growth factors. Bar graph representing the densitometry of spotted antibody array results. Data are represented as the mean spot pixel density subtracted from the averaged background signal.

A preliminary analysis of the CM secretome was performed using a high-density protein array. Antibody arrays was performed on 3 distinct CM samples. CM contained 21 of the 111 proteins assayed (Figure 5), which included a broad spectrum of molecules involved in cellular growth, differentiation, gene expression, migration, immunity, and inflammation.

## 4. Discussion

The present study demonstrated that a single CM treatment was able to reverse the behavioral neuropathic pain and to modify cytokine signaling associated with its maintenance. Importantly, the profile and magnitude of the CM-induced beneficial effects were similar to those induced by MSCs transplantation, highlighting the potential of this cell-free therapeutic approach for the treatment of neuropathic pain.

The MSCs transplantation induced a robust and long-lasting antinociceptive effect in neuropathic mice. In fact, the consistent antinociceptive properties of mesenchymal stem cells on neuropathic conditions have been demonstrated [19–23]. Recent evidence indicate that MSCs may act as biologic “pumps” by releasing antinociceptive molecules that act in the pain processing centers or the sites of injury in order to mediate their therapeutic effects on neuropathic pain [34]. The present study reinforces this hypothesis, since the MSC-induced antinociceptive effect started 24 hours after transplantation, a very short time for induction of neural repair. Pain-like behavior data from CM-treated mice are also in line with this idea, considering that CM displayed marked antinociceptive effects with magnitude similar to MSCs. Regarding the effects profile, both the onset of the antinociceptive effect and the plateau of this effect are later for MSCs than MC. This profile seems to reflect a predictable kinetic pattern. Following intravenous administration, CM bioactive substances are readily bioavailable to act on their tissue targets, inducing an immediate antinociception. On the other hand, injected MSCs are distributed throughout the body, retained in filter organs, and migrate to injured tissues days after administration [35], where they secrete bioactive substances, inducing later antinociception.

Since experimental studies have shown that the paracrine action of stem cells, rather than their transdifferentiation, accounts for the functional restoration of damaged tissues, the development of cell-free therapeutics based on secretome from stem cells has been explored. Over the last years, a growing number of studies have demonstrated that the conditioned medium from mesenchymal cell cultures contains a large variety of cytokines, enzymes, and growth factors, promoting beneficial effects in different experimental conditions. The therapeutic efficacy of conditioned medium of MSCs has also been successfully demonstrated in animal models of neurological disorders [14, 16]. Recently, Brini and coworkers have demonstrated that CM of human mesenchymal cells from adipose tissue controls complications of experimental diabetes, such as neuropathic pain, skin innervation loss, and Th1/Th2 unbalance [35]. In the present study, the therapeutic potential of CM on posttraumatic painful neuropathy was demonstrated for the first time. Corroborating the neuroprotective properties attributed to conditioned medium, the antinociceptive effect induced by a single injection of CM was not reversed throughout the evaluation period, an effect that is not reached by analgesic drugs. In fact, the antinociceptive effect of gabapentin, the gold standard to clinical control of neuropathic pain, was completely reverted 12 h after administration. These data indicate that, in addition to an early release of antinociceptive molecules, CM induces disease-modifying effects.

Interestingly, the antinociceptive effect induced by single CM injection was as long-lasting as that induced by MSCs. Brini and coworkers described a similar phenomenon in diabetic neuropathy [35]. It has been suggested that, during neuropathy, mesenchymal stem cells induce reparative effect on the nervous tissue mainly through paracrine support of injured cells, regulation of immune response, and local progenitor cell proliferation and differentiation [36]. It is possible to propose that the long-lasting MSC-induced antinociception is a reflex of this reparative effect instead of a continuous secretory action, considering that frequently MSCs present poor cellular retention within the injury environment [37] and the present results showing the CM persistent effects. Likewise, bioactive substances from CM can modulate the immune and neuronal environments, triggering or repressing signaling events that modify the course of neuropathy.

While several mechanisms have been implicated in the development of neuropathic pain, it is now clear that the neuroimmune response triggered by the neuronal damage is a key event of this painful syndrome [38]. In response to neural injury, neurons, glia, and immune cells that participate in the response to injury produce cytokines at peripheral and central sites. The cytokine signaling appears to start with the production of key cytokines, such as IL-1*β* and TNF-*α*, that produce direct effects on sensory neurons and coordinate the subsequent production of further downstream cytokines and numerous other hyperalgesic mediators [39]. Moreover, converging lines of evidence have indicated that IL-6 plays a critical role in the pathogenesis of neuropathic pain [40].

Upregulation of IL-1*β*, TNF-*α*, and IL-6 at multiple levels of the neuroaxis represents a common event of different neuropathic pain syndromes [39–42]. In line with this concept, in the present study following the PSL, there was upregulation of L-1*β*, TNF-*α*, and IL-6 in both the spinal cord and sciatic nerve. Importantly, CM treatment inhibited the IL-1*β*, TNF-*α*, and IL-6 upregulation in the neuroaxis, in parallel with its effects on pain-like behaviors. In addition, CM was able to enhance the local and spinal levels of IL-10 in neuropathic mice. These modulatory properties were also displayed by the MSC transplantation. Corroborating the present data, treatment of diabetic mice with human mesenchymal cells from adipose tissue or their secretome was able to modulate pro/anti-inflammatory cytokine levels [35]. Numerous experimental data provide evidence that proinflammatory cytokines induce neuropathic pain. Whereas direct application of exogenous TNF-*α*, IL-6, or IL-1*β* induces pain, blockade of these cytokines using neutralizing antibodies, antagonists, or similar strategies inhibits the development of neuropathic pain behavior [42]. Anticytokine agents, successfully used for many years for the clinical control of inflammatory pain, have been investigated for clinical efficacy in neuropathic pain states [43, 44]. However, their use may be limited by the redundancy of the cytokine system, in which blockade of one cytokine is frequently compensated by upregulation of others with similar effects. To overcome this obstacle, the therapeutic use of the anti-inflammatory cytokine IL-10 have been proposed. IL-10 is neuroprotective and one of the most powerful endogenous counter-regulators of proinflammatory cytokine function that acts in the nervous system. A large body of evidence supports a therapeutic role for IL-10 in suppressing neuropathic pain [45]. Clinical data showing that patients with chronic pain present suppressed IL-10 functions corroborate this idea [41, 46]. On the other hand, the short-term efficacy of protein injections, which corresponds to the IL-10 half-life of protein, limits this approach to treat chronic conditions like neuropathic pain. Considering the above-described scenario, it has been proposed that the ideal cytokine-based treatment of neuropathic pain consists of the combined inhibition of proinflammatory cytokines or shifting the balance towards anti-inflammatory cytokines [42, 47]. Importantly, the present data showed that CM treatment induce an antinociceptive effect associated with a reestablishment of the balance between pro- and anti-inflammatory cytokines in the peripheral and central nervous system. Although the modulatory effect of MSCs on cytokines expression during neuropathic conditions has been previously demonstrated and proposed as a mechanism of cell-induced antinociception [47–49], the present work shows that a CM preparation of MSCs also presents this modulatory property.

Aiming to understand the molecular mediators of the CM-induced antinociceptive effect, a preliminary analysis of the CM secretome was performed. Interestingly, although the antibody array revealed the presence of several cytokines in CM, IL10 was not detected. This result indicates that CM is not the primary source of IL-10 but stimulates the local release of this cytokine in the nervous system during neuropathy. Among the 21 proteins presented on CM, some of them, such as hepatocyte growth factor (HGF), vascular endothelial growth factor (VEGF), chemerin, and angiopoietin-1, are potential mediators of the antinociceptive effects exerted by the CM on neuropathic conditions. Besides its prominent role in angiogenesis, VEGF also exerts important neuroprotective effect during neuropathic conditions [50]. The complex role of VEGF on pain modulation has been demonstrated through clinical and experimental approaches. Clinical neutralization of VEGF-A with anti-VEGF-A therapies or VEGF-A receptor inhibitors induce pain [51, 52]. Moreover, neutralization of endogenous VEGF-A increased pain sensitivity in a model of chemotherapy-induced neuropathy [53], while treatments with VEGF induce antinociceptive effects in experimental painful conditions [50, 54, 55]. On the other hand, VEGF has also been shown to induce pronociceptive effects [56, 57]. This dual effect has been explained by different actions of the distinct VEGF isoforms. The isoforms VEGF-A165a and VEGF-A165b have opposing effects on pain, while VEGF-A165a is pronociceptive, whereas VEGF-A165b exhibits antinociceptive properties [54]. Considering the evident modulatory role of VEGF on pain, it is possible to correlate the high levels of this growth factor detected in CM to its antinociceptive effect. On the other hand, overexpression of VEGF has been related with serious side effects, such as increased risk of tumor formation [58, 59]. Likewise, the tumorigenic potential of stem cells has been associated with their ability to continuously secrete angiogenic factors, such as VEGF [60]. In the present study, the pain-like behaviors were reversed by a single CM treatment. Considering the short biological half-life of VEGF, it is possible to propose that this cell-free therapy is a strategy with greater translational potential than the cellular transplantation for the clinical control of neuropathic pain.

Moreover, chemerin and angiopoetin-1 should also be highlighted, considering their beneficial effects on experimental neuropathic pain. The antinociceptive effect of chemerin was previously demonstrated in a mouse model of neuropathic pain [61]. Angiopoetin-1, an endothelial cell growth factor, displays antiapoptotic, angiogenic, and neurotrophic properties on neurons of the central and peripheral nervous system [62–64]. In a mouse model of diabetic peripheral neuropathy, angiopoietin-1 promoted angiogenesis, suppressed inflammation, and induced signs of regeneration in sciatic nerves [65], pointing angiopoietin-1 as a new treatment option to improve neuropathy.

HGF is also a good candidate to be one of the factors facilitating CM-induced therapeutic effects on neuropathy because of its powerful angiogenetic and neurotrophic actions [66, 67] and its clinical therapeutic effects in patients with painful diabetic neuropathy [68]. Tsuchihara et al. demonstrated that HGF reduces behavioral pain and morphologic alterations in sciatic nerve of neuropathic rats [69]. In addition, the HGF-induced antinociception was concomitant with a reduction of the IL-6 upregulation in the sciatic nerve of neuropathic rats, similarly to the result obtained here with the CM treatment.

## 5. Conclusions

The strategy that supports the proposal of replacement of the current pharmacological treatments by cellular therapies in neuropathic pain is a possible modulatory action on the pathophysiology of neuropathy. This property may confer to the cell therapy longer lasting, or even curative, effects in detriment to the palliative effects of the available analgesics. In the present study, the CM treatment induced a long-lasting antinociceptive effect with a disease-modifying profile similar to that showed by MSCs. This comparative study opens a new perspective for the treatment of neuropathic pain since it demonstrates that it is possible to develop cell-free treatments that are able to retain the benefits of cell therapy for neuropathic pain without exhibiting the inherent difficulties of cell-based therapy.
